# Observations on the Extraction of Teeth, and the Instruments in Use for That Purpose

**Published:** 1844-07-27

**Authors:** J. F. B. Flagg

**Affiliations:** Surgeon Dentist; Philadelphia


### OBSERVATIONS ON THE EXTRACTION OF TEETH, AND THE INSTRUMENTS IN USE FOR THAT PURPOSE.

By J. F. B. Flagg, M. D. Surgeon Dentist.

Many diseases of the teeth, and of their contiguous parts, render a removal of them absolutely necessary, in order to secure the return to healthy action of the portion affected, as well as to afford relief from pain. In consideration of this, it becomes an important desideratum as to the most *scientific, expeditious and least painful* method of performing this operation.

Errors prevail upon this subject, both in regard to the instruments in general use, and the manner of their application. Besides, there are some moral considerations too often lost sight of by practitioners of the dental art. It is wrong to attempt the extracting of a tooth for a child, when he is assured "the doctor is only going to look at it," notwithstanding you may be encouraged to do so by a whisper or a knowing wink on the part of its parent or guardian; and it is equally a *mistake* to tell the confiding little sufferer that he is not to be hurt in the least, when nine times in ten, the persons so attempting to inspire courage, will either leave the room, or show by some other obvious sign that *their* nerve is not equal to the witnessing so much torture. It is likewise a very great *error* to believe that you are about to have your tooth extracted with the thumb and finger, when the operator has a concealed instrument in his sleeve.

These may appear trifling in themselves, but when considered in connection with the prevailing abhorrence of the adult to undergo this necessarily somewhat painful operation, is it not reasonable to attribute much of their fear to the method of their treatment as children.

I have been led to the foregoing remarks, more from their applicability to the subject of extracting teeth generally, than from any particular bearing they may have upon the relative merits of the various instruments used for this purpose; a few of which I will proceed to consider, and respectfully submit to the attention of your readers.

As it would not be even amusing to describe many of the ancient instruments applied to remove pain, as it were, by *counter irritation*, except to those curious in the inventions of inquisitorial tortures, I will pass over such, and proceed at once to the consideration of a few of those in most common use at the present day.

The *German Key* has been used more generally, for many years past, than probably all other instruments combined. It has been subjected to various modifications or *improvements* according to the genius or fancy of the maker or operator; few of which, however, have been found to possess any real advantages over the original invention. For example; the *Key* which most prevails in this country, is that with the irregular or crooked shank, so constructed for the purpose of avoiding the cutting edges of the front teeth, when the fulcrum is placed *within* the arch of the jaw, and the tooth is removed by turning it *in*. Now a correct knowledge of the anatomy of these parts compel all scientific dentists to turn the tooth in an opposite direction. There are rarely exceptions to this general rule; it sometimes happens, that, from a crowded state of the tooth to be removed, there may not be sufficient space to allow of its passing *outward*; in this case we are justified in turning it as nature seems to dictate; taking sufficient care, however, to load the fulcrum with many bandages, in order to prevent its falling below the neck of the tooth, and to cause the pressure upon the more elevated portion of the jaws and teeth. In a proper application of the instrument, under these circumstances, it will be perceived that the matter of any other *turn* in the instrument than that which is required to remove the tooth is entirely uncalled for. With a judicious selection of the hook, and a proper enlargement of the fulcrum, by means of bandages, this is a safe and useful instrument in the hands of the operator.

The *Graver* or *Punch* has been in use for the removal of stumps or roots, and occasionally entire teeth, for several years; many, otherwise good operators, will not trust themselves with the use of this instrument, deeming it unsafe, but in the hands of the skilful it becomes too important ever to be *meanly concealed*.

The only remaining instruments to which I would ask attention, are the *Extracting Forceps;* and to those only which are formed with strict reference to their legitimate purposes. The forceps best calculated to remove a tooth, are those which will grasp the neck of the tooth most effectually, even below the *alveoli;* and in order to effect this, it is necessary to have one or more pairs adjusted to every variety of teeth, both superior and inferior. Great care is required, as well in acquiring the proper hold of a tooth, as in effecting the removal with these instruments; otherwise serious mischief may result, not only to the tooth in question, but others may be equally jeoparded. The following case will serve to illustrate:—

Mr. D. R. of New Bedford, Mass. called upon me to remove the roots of the first inferior molar, which had been broken in consequence of injudicious application of the forceps; and this was not the whole extent of the mischief he suffered; as, for want of entire control, the operator allowed the instrument to rebound against the upper central incisor with sufficient force to break that also. Such *accidents*, I am sorry to say, are as often the result of ignorance as carelessness in the profession.

If properly used, forceps possess decided advantages over all other instruments, frequently causing the patient to exclaim "*it did not hurt;*" "I *thought* it would slip off." When, in fact, the tooth was out; and similar expressions to the foregoing, are of daily occurrence, fully confirming the comparative ease and comfort in their proper application. The most prominent advantage these instruments possess over the *key* is, the avoiding the necessary pressure upon the surrounding soft, and not unfrequently inflamed portions of the jaw, which *pressure*, in most cases, becomes more serious cause of complaint than dismemberment itself.

I will close this article, by relating the following anecdote, which shows the effect often produced by the use of the keys. Some years ago, while on a visit to the Catskill Mountains, seeking relief from care and the oppressive atmosphere of a crowded city, I chanced to meet with a young and well educated Irish gentleman, but recently arrived from the "old country;" his address was pleasing, but he had one very awkward gesticulation of constantly carrying his hand to the mouth, and seizing violently what proved in the sequel to be an offending tooth. Having thrown into my trunk an extracting key, to meet similar emergencies, I ventured to suggest that it would afford me *peculiar pleasure* if I were sufficiently in his confidence to be allowed to officiate; in this I was successful; he seated himself in the barber's chair and every preliminary was arranged.

For the information of those who have never visited this spot, it may be well to give a brief description of its location, the better to appreciate what follows. The mountain house is a very convenient edifice, with some pretensions to classical elegance even in its front, which looks directly upon the waters of the Hudson, from a distance, varying from twelve to thirty miles; and at an elevation of about 3000 feet. It stands upon an abrupt ledge of rocks or precipice; and immediately at its western wing stands an aged but storm-stricken tree, which has acted many times the part of lightning-rod or protector to the house. A short distance, somewhat in the rear of the buildidg, the hill rises some 300 feet higher, and completely destroys the prospect in that direction. Now it is not unfrequently the case that a cloud arises, and comes around this elevation so suddenly as to give no previous intimation of its existence, and you become immediately deluged in rain. Such was the case upon the occasion alluded to. Having placed, *secundum artem*, the key upon the tooth, I began to pull: at that instant, the lightning's flash and one report of thunder were simultaneous; the tree, which stood almost at our feet, became shivered, the instrument appeared to be made of *fire;* one nervous twitch on my part, and the tooth was out. "Heavens!" I exclaimed, "what a clap of thunder!" The patient arose, and immediately responded, with that brogue so peculiar to the inferior portion of his countrymen, "THUNDER? *I thought it a part of the operation!*"

No greater evidence, I think, can be adduced to prove the superiority of the forceps over the key, than the fact that some *operators* are having constructed a mongrel instrument, resembling the forceps in the formation of the *handle;* but upon the opposite extremity is placed the *hook and fulcrum* of the key; one-half of which instrument is intended for the *eye* of the patient, and the other for the *mouth.*

*Philadelphia, July* 3, 1844.

